# CaP Nanoparticles Improve the Effect of dsRNA on Gene Expression, Growth, and Mycotoxin Production of Toxigenic *Fusarium graminearum*

**DOI:** 10.3390/ijms262010021

**Published:** 2025-10-15

**Authors:** Alexander A. Stakheev, Polina Bagdasarova, Eugene A. Rogozhin, Victoria Tikhomirova, Ekaterina Popova, Assel Akhmetova, Olga Kost, Natalia O. Kalinina, Michael Taliansky, Sergey K. Zavriev

**Affiliations:** 1 M.M. Shemyakin and Yu.A. Ovchinnikov Institute of Bioorganic Chemistry, 117997 Moscow, Russia; polina44815283v@gmail.com (P.B.); rea21@list.ru (E.A.R.); vetikhomirova@gmail.com (V.T.); popova.ekaterina1995@gmail.com (E.P.); olga.a.kost@gmail.com (O.K.); kalinina@belozersky.msu.ru (N.O.K.); michael.taliansky@mail.ru (M.T.); szavriev@ibch.ru (S.K.Z.); 2 Faculty of Biology and Biotechnology, National Research University Higher School of Economics, 101000 Moscow, Russia; 3 Chemistry Faculty, Lomonosov Moscow State University, 119991 Moscow, Russia; 4 Physical Faculty, Lomonosov Moscow State University, 119991 Moscow, Russia; akhmetovaai@my.msu.ru; 5 A.N. Belozersky Institute of Physico-Chemical Biology, Lomonosov Moscow State University, 119992 Moscow, Russia

**Keywords:** *Fusarium graminearum*, RNA interference, spray-induced gene silencing, CaP nanoparticles, *FgVe1* gene, deoxynivalenol, gene expression, plant protection

## Abstract

*Fusarium* species and the mycotoxins produced by them represent a significant problem for agriculture and human health. Thus, the development of novel management strategies and tools is of high importance. Spray-induced gene silencing (SIGS), based on the natural mechanism of RNA interference (RNAi), has been considered as a highly specific and ecologically safe alternative to chemical fungicides, the use of which is restricted by the emergence of resistant strains and environmental concerns. At the same time, massive application of SIGS is challenged by the degradability of RNA molecules in the environment. Nanoparticles have been widely applied to protect RNA from degradation and improve its action. The aims of this study were to evaluate whether RNAi-mediated silencing of the regulatory *FgVe1* gene leads to inhibition of growth, mycotoxin production, and pathogenicity of *Fusarium graminearum* and whether the use of CaP nanoparticles (CaPs) as double-stranded RNA (dsRNA) carriers enhances and prolongs the silencing effect. It was shown that dsRNA treatment of fungal liquid cultures resulted in 19.78-fold silencing of *FgVe1* expression as well as inhibition of expression of genes related to secondary metabolism, including those involved in trichothecene and aurofusarin biosynthesis, thus leading to a reduction in DON accumulation and changes in culture color. The results also demonstrated that naked dsRNA and CaPs:dsRNA nanocomplexes differed in their abilities to induce a high silencing effect at different time points. Naked dsRNA proved more effective in inducing silencing in the early stages of fungal growth, whereas application of nanocomplexes provided a prolonged effect up to 10 days in liquid cultures and up to 14 days on detached leaves. The obtained data can be considered as a basis for the further development of new efficient SIGS-based plant protection strategies.

## 1. Introduction

Fusarium head blight (FHB), or scab, is one of the most harmful diseases of cereal crops, leading to annual losses estimated at several billion USD [1,2,3]. Besides having a direct impact on grain quality and quantity, FHB agents produce a wide range of mycotoxins, including trichothecenes, fumonisins, and zearalenone, that can accumulate in the food chain and cause serious diseases in humans and livestock [4,5,6,7]. *Fusarium graminearum* Schwabe has been considered the most devastating fungal pathogen of agricultural plants in the world [8,9]. The main mycotoxins produced by this fungus are deoxynivalenol (DON), nivalenol (NIV), and their acetylated derivatives 3-acetyldeoxynivalenol (3-ADON), 15-acetyldeoxynivalenol (15-ADON), and 4-acetylnivalenol (4-ANIV). DON is a potent inhibitor of protein biosynthesis in mammals and an important fungal virulence factor towards a host plant [10,11,12,13]. Recent studies have demonstrated that the rate of DON contamination of cereal grains in Europe and other parts of the world is about 50% or even higher [14,15]. Therefore, efficient means for the management of plant diseases caused by mycotoxigenic fungi are of high importance. Fungicides are still the most commonly used approach, but their wide application has resulted in pathogen resistance development [16,17]. Moreover, chemicals, especially in high concentrations, pose a serious risk to human and animal health [18].

Methods based on targeted silencing of a pathogen essential gene(s) via RNA interference (RNAi) are an ecologically safe and highly specific strategy of plant protection. RNAi is a natural mechanism regulating gene expression in eukaryotes, which was first discovered in the nematode *Caenorhabditis elegans* Maupas, 1899 [19]. Since then, RNAi has been described in many other organisms and has become a powerful tool for basic and applied research. In plant biology, RNAi-based approaches have been used for gene function analysis, improving abiotic stress tolerance and crop quality, as well as pathogen and pest control [20,21,22,23,24]. Generally, there are two strategies of RNAi-based protection against pathogens: host-induced gene silencing (HIGS) based on transformation of the host plant followed by expression of a double-stranded RNA (dsRNA) that silence a pathogen gene(s), and spray-induced gene silencing (SIGS) using direct application of a specific dsRNA onto the pathogen or plant surface [25]. HIGS is an efficient but technically challenging technique, facing legislative restrictions in countries, which prohibits the development of genetically modified organisms. The use of SIGS allows genetic modifications and time-consuming manipulations to be avoided, making this technique the most promising tool for targeted dsRNA delivery and management of pathogens. After the pioneering work of Koch et al., 2016 [26], SIGS has been used against mycotoxin-producing *Fusarium* species (reviewed in [27,28]). In these studies, dsRNAs were applied in a naked, unprotected form that can be easily degraded under environmental conditions [29]. Thus, the development of methods of dsRNA protection from degradation and increasing the duration of its action is an important task that needs to be solved to introduce dsRNA-based pesticides into agricultural practice.

The use of nanocarriers is one of the prospective ways to improve the stability of dsRNA and prolong its effect. To date, a number of works describing the application of dsRNA–nanoparticle complexes to combat different plant pathogens have been published [30,31]. Particularly, layered double hydroxide clay (LDH) and chitosan were successfully employed for RNAi-based assays against pathogenic fungi [32,33,34]. At the same time, the search for cheap, efficient, and safe nanocarriers is still ongoing. Calcium phosphate nanoparticles (CaPs) have become widely used in medicine due to their biocompatibility and biodegradability, as well as having a strong affinity to therapeutic drugs [35,36,37]. Efficient binding of CaPs to proteins and nucleic acids and targeted delivery of the complexes to tissues and cells have also been shown [38,39]. In agriculture, CaPs have been used as fertilizer itself [40,41] or as a component of complexes promoting plant growth and other valuable traits [42,43]. However, the application of CaPs as dsRNA carriers for RNAi-based management of plant pathogens, including toxigenic fungi, has not yet been described.

Selection of an appropriate gene to be silenced is one of the most important issues to be solved for an effective RNAi-based assay. In fungi, potential target genes encode transcription regulators, enzymes of mycotoxin biosynthesis, fungicide targets, components of RNAi machinery, and pathogenicity factors [26,44,45,46,47,48]. Genes encoding components of the fungal-specific Velvet complex, which includes VE1, VELB, and LAEA proteins, are among the most promising targets for silencing. The Velvet complex is responsible for fungal response to light, also participating in the regulation of such processes as sexual development, fruit body formation, and secondary metabolism [49]. VE1, which is encoded by the *FgVe1* gene in *F. graminearum*, represents a conserved protein, and its homologs were found in different *Fusarium* species and other fungal genera. In *Aspergillus* species, such as *A. flavus* and *A. parasiticus*, *VeA* positively regulates aflatoxin and sterigmatocystin biosynthesis [50,51]. In *F. verticillioides*, *FvVe1* deletion completely suppresses fumonisin biosynthesis via inhibiting expression of the *Fum21* gene, encoding the pathway-specific regulator [52]. ∆*FgVe1* strains of *F. graminearum* were characterized by significantly reduced DON production, hyphal formation, conidiation, and virulence [53]. Moreover, *FgVe1* deletion resulted in the abolished biosynthesis of red pigment aurofusarin; thus, mutant strains exhibited a change of culture color [54]. In closely related *F. pseudograminearum*, knockout of the homologous gene leads to the loss of pathogenicity towards wheat [55]. In a widespread pathogen *F. oxysporum*, *VeA* governs virulence and biosynthesis of a depsipeptide mycotoxin beauvericin [56]. Therefore, *FgVe1* can be considered an appropriate target for RNAi.

The hypothesis of this study was that dsRNA-mediated silencing of *FgVe1* affects *F. graminearum* growth, DON production, and pathogenicity on detached wheat leaves, as well as that the application of CaPs as dsRNA carriers improves the effect. To elucidate this, the silencing effects of three dsRNAs targeting the *FgVe1* gene were analyzed. Then, the most efficient dsRNA was used in naked form as well as in complex with CaPs to inhibit the expression of several genes involved in secondary metabolism, as well as to reduce DON accumulation in liquid media. Spraying of both naked dsRNA and CaPs:dsRNA nanocomplexes was also applied to control *F. graminearum* on detached wheat leaves. The results provide a new opportunity for improved management of FHB agents and mycotoxin contamination of plant material.

## 2. Results

### 2.1. Silencing Effects of Different dsRNAs and Selecting an Optimal Concentration

**Figure 1:**
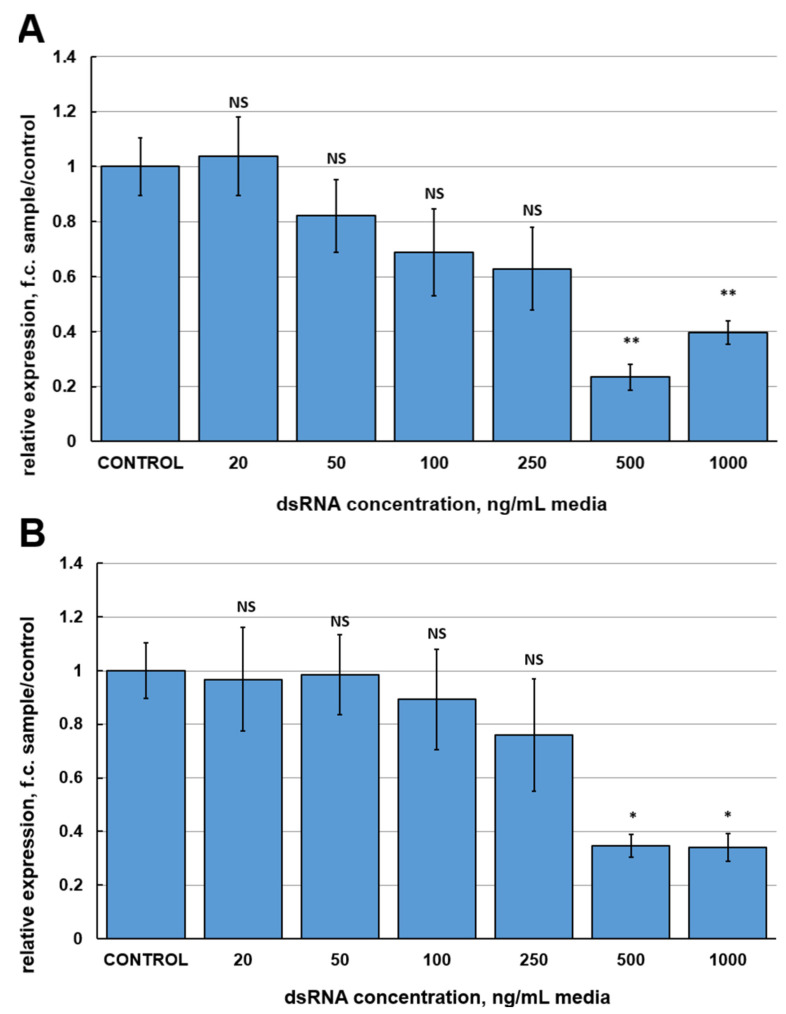

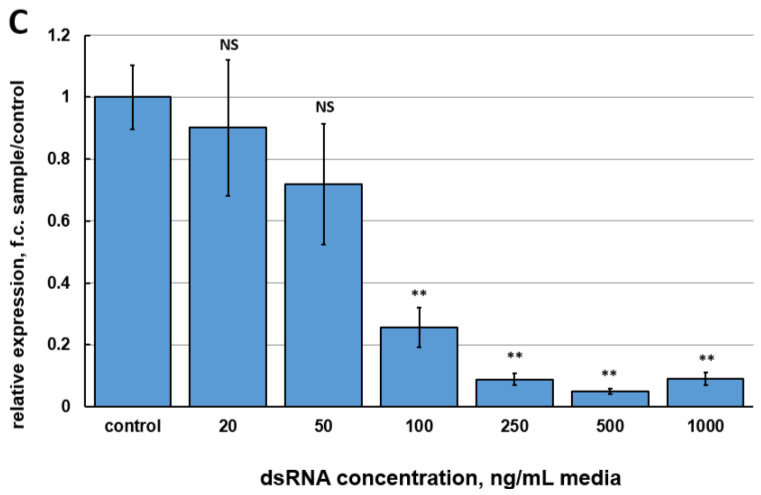
Relative expression levels of the *FgVe1* gene (fold changes, f.c.) in liquid cultures of *F. graminearum* MFG 58918, treated with different concentrations (20, 50, 100, 250, 500, and 1000 ng/mL of media) of three candidate dsRNAs: FgVe1_150 (**A**), FgVe1_370 (**B**), FgVe1_540 (**C**). The experiment was carried out in four biological replicates with two technical replications for each sample. The *FgVe1* expression level for the control was considered as 1.0. The *Tef1α* gene was used as a reference. Bars represent mean ± SD (n = 4). Symbols indicate the significance levels of differences according to the Tukey HSD test: ** *p* < 0.01; * *p* < 0.05; NS—no significant difference.

The purpose of the first series of experiments was to estimate the efficiencies of different dsRNAs to silence the *FgVe1* gene. To obtain candidate dsRNAs, three fragments of the *FgVe1* gene were selected. Two of these fragments (150 and 540 bp) are located at the 5′-end of the gene, whereas another fragment (370 bp) is located in the middle of the coding sequence (Figure S1). The range of concentrations to compare was from 20 to 1000 ng per mL of media. Relative gene expression analysis followed by ANOVA statistical test demonstrated that only two (500 and 1000 ng/mL) out of six concentrations of FgVe1_150 and FgVe1_370 dsRNAs induced statistically significant inhibition of *FgVe1* gene expression (Figure 1A,B). Treatment with 500 and 1000 ng/mL of FgVe1_150 dsRNA resulted in 4.267- (*p* = 0.00086) and 2.524-fold (*p* = 0.00479) down-regulation of the *FgVe1* gene, respectively. In samples, treated with FgVe1_370 dsRNA, the target gene was 3.018- (500 ng/mL, *p* = 0.018) and 3.062-fold (1000 ng/mL, *p* = 0.021) down-regulated. FgVe1_540 dsRNA proved to be the most efficient variant, providing strong silencing at a concentration range from 100 to 1000 ng/mL (Figure 1C). The most significant effect was achieved at 500 ng/mL: down-regulation of the *FgVe1* expression was 19.78-fold changed (*p* = 0.00021). Interestingly, the minimal concentrations tested (20 and 50 ng/mL) had no statistically significant effect on *FgVe1* expression regardless of which dsRNA was applied. Another important fact is that the highest concentrations did not provide the maximum effect. Based on the obtained results, the FgVe1_540 dsRNA was chosen for further studies.

### 2.2. Characterization of CaP Nanoparticles and CaPs:dsRNA Nanocomplexes

**Figure 2:**
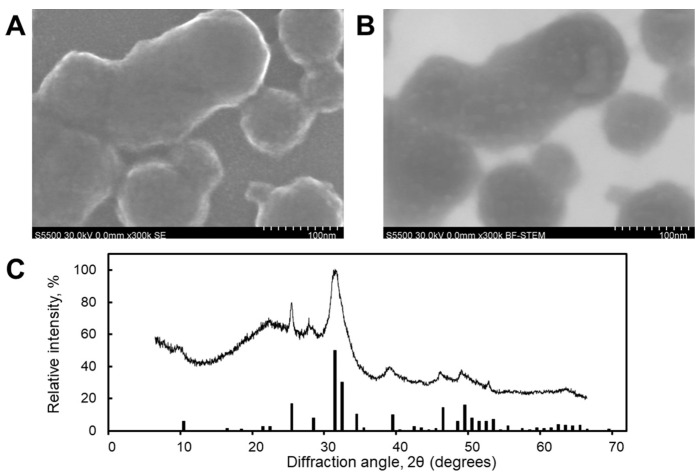
SEM (**A**) and STEM (**B**) images and X-ray diffractogram (**C**) of CaPs. The bars present the peaks assigned to the characteristic spectrum of hexagonal hydroxyapatite.

Synthesized CaP nanoparticles (CaPs) were characterized by scanning electron microscopy (SEM) (Figure 2A) and scanning transmission electron microscopy (STEM) (Figure 2B). The CaPs were characterized by a rounded shape with a particle size distribution in the range of 20–150 nm (Figure 2A,B). The STEM image also reveals the porous structure of the CaPs (Figure 2B).

Results from dynamic light scattering (DLS) showed an average hydrodynamic diameter of 80 ± 20 nm (the polydispersity index (PDI) was 0.19), consistent with the particle sizes measured from SEM and STEM images. The ζ-potential was −25 ± 2 mV. Phase analysis indicated that the CaPs were a mixture of hexagonal hydroxyapatite (Ca_10_(PO_4_)_6_(OH)_2_) and amorphous calcium phosphate (Caₓ(PO_4_)ᵧ·zH_2_O). The presence of the amorphous phase was deduced from the broadening of the diffraction peaks associated with crystalline hydroxyapatite (Figure 2C).

The CaPs exhibited significant stability when stored as aqueous suspensions; their size and ζ-potential remained unchanged throughout a one-month period at 4 °C.

**Figure 3:**
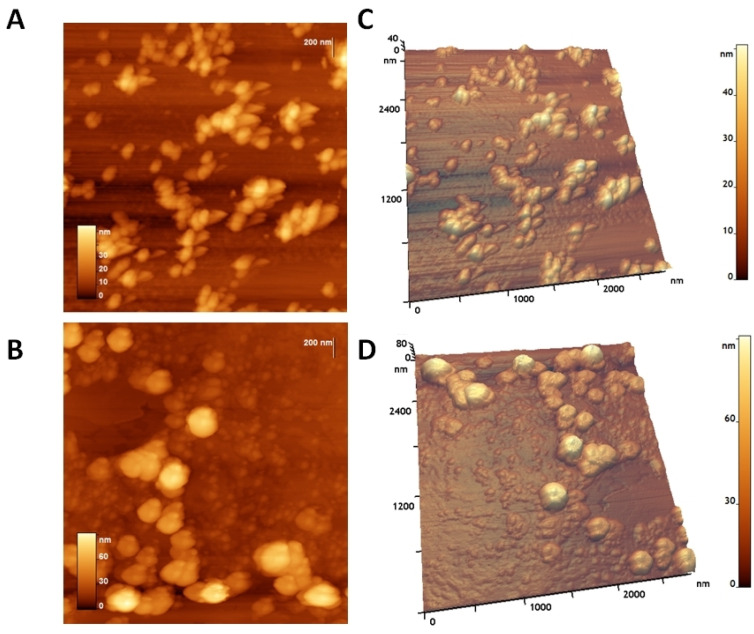
AFM of CaPs on graphite (top images) and CaPs:FgVe1_540 dsRNA nanocomplexes on graphite (bottom images): (**A**,**B**) 2D image; (**C**,**D**) 3D image.

Atomic force microscopy (AFM) showed that the CaPs were predominantly spherical (Figure 3A,C). Particles were not prone to destruction when exposed to a cantilever and were quite elastic and did not aggregate; when drying on graphite or mica, they tend to form a monolayer, but a double layer can also be seen. The typical diameter was 35 ± 7 nm. AFM showed a lower diameter than SEM due to adsorption on the substrate while drying, and due to the form of the cantilever.

The addition of dsRNA significantly altered the morphology of the CaPs particles. The dsRNA-loaded particles underwent a shape transformation, resulting in an increase in their volume and diameter. The roughness of the particles and the height increased nearly twice. Small spheres are visible on the substrate and on the particles themselves. These spheres, with a height of about 6–10 nm, likely belonging to dsRNA, were visible on the surface of CaPs (Figure 3B,D). Thus, CaPs readily form complexes with dsRNA, which also remain stable on the substrate and do not disintegrate upon drying. AFM images of the adsorption of CaPs on F. graminearum can be found in Supplementary Figure S3.

### 2.3. Evaluation of the Effect of Naked dsRNA and CaPs:dsRNA Nanocomplexes on F. graminearum Gene Expression at Different Time Points

**Figure 4:**
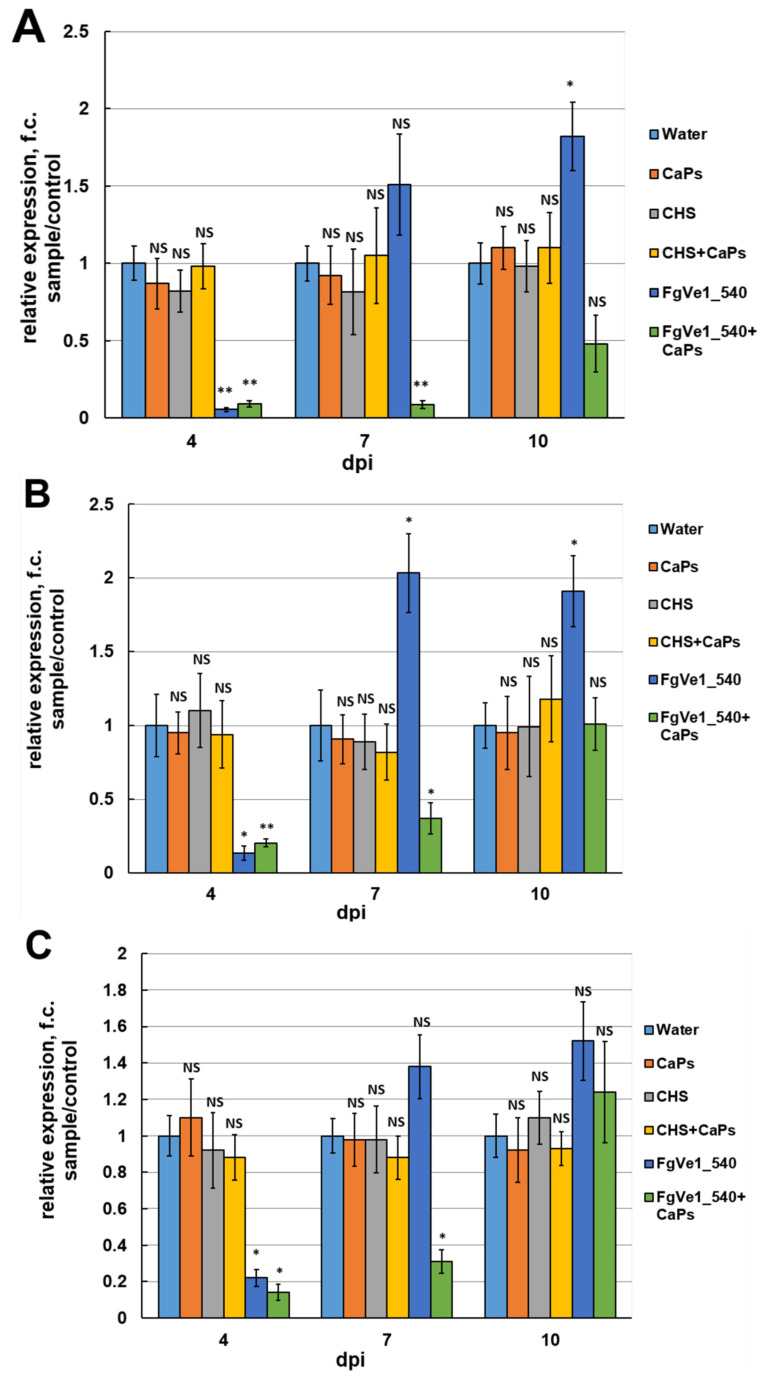

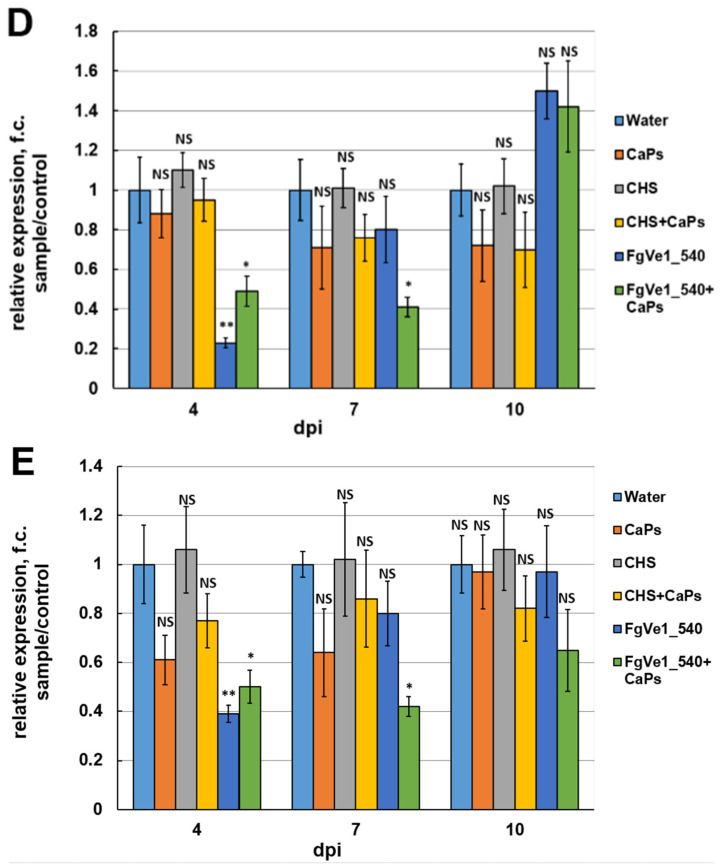
Effect of FgVe1_540 dsRNA (12.5 ng/mL) and CaPs:FgVe1_540 (62.5: 12.5 ng/mL) dsRNA nanocomplexes treatment on the relative expression of the *FgVe1* (**A**) and four genes related to fungal secondary metabolism: *Tri5* (**B**), *Tri6* (**C**), *Pks12* (**D**), *AurR1* (**E**), in liquid cultures of *F. graminearum* MFG 58918. Analyses were carried out at 4, 7, and 10 dpi. Control treatments included (i) sterile water (expression levels of target genes in these samples were considered as 1.0); (ii) CaPs suspension (62.5 ng/μL); (iii) ChS dsRNA (12.5 ng/μL); (iv) CaPs:ChS dsRNA nanocomplex (62.5: 12.5 ng/μL). The experiment was carried out in four biological replicates with two technical replications for each sample. The *FgVe1* expression level for the control was considered as 1.0. The *Tef1α* gene was used as a reference. Bars represent mean ± SD (n = 4). Symbols indicate the significance levels of differences according to the Tukey HSD test: ** *p* < 0.01; * *p* < 0.05; NS—no significant difference.

Naked FgVe1_540 dsRNA and CaPs:FgVe1_540 dsRNA nanocomplexes were then used for treatment of liquid cultures to answer the following questions: (i) Whether silencing of the *FgVe1* gene leads to down-regulation of expression of the genes responsible for secondary metabolism, e.g., *Tri5*, *Tri6* (trichothecene biosynthesis), *Pks12,* and *AurR1* (aurofusarin biosynthesis)? (ii) Does it result in DON accumulation inhibition? (iii) Can CaPs enhance and prolong the silencing effect of dsRNA? Besides naked specific dsRNA and CaPs:dsRNA nanocomplexes, the following controls were used: sterile water, CaPs suspension, non-specific dsRNA targeting the *ChS* gene of hop (*Humulus lupulus* L., Figure S2) (ChS_dsRNA), and CaPs:ChS_dsRNA nanocomplexes. Relative expression of the studied genes was assessed at different time points: 4, 7, and 10 days post-inoculation (dpi). It is noteworthy that none of the control samples demonstrated a statistically significant difference in *FgVe1* relative expression in comparison to the samples treated with sterile water. Therefore, CaPs or non-specific dsRNA did not have any effect on *FgVe1* gene expression. At 4 dpi, naked FgVe1_540 dsRNA demonstrated more efficient silencing of *FgVe1* expression than CaPs:FgVe1_540 dsRNA nanocomplex: 18.54- (*p* = 0.0005) and 11.1-fold (*p* = 0.00066) down-regulation, respectively (Figure 4A). As expected, silencing of *FgVe1* gene resulted in suppressed expression of the genes of secondary metabolism: *Tri5* (7.4- (*p* = 0.04) and 4.9-fold (*p* = 0.0068) down-regulation under naked dsRNA and nanocomplex, respectively, Figure 4B); *Tri6* (4.54- (*p* = 0.024), and 5.56- (*p* = 0.032) fold, Figure 4C); *Pks12* (4.34- (*p* = 0.0072), and 2.04- (*p* = 0.016) fold, Figure 4D); and *AurR1* (6.23- (*p* = 0.009), and 2.0- (*p* = 0.034) fold, Figure 4E). Therefore, all these genes, except *Tri6*, were more efficiently silenced by treatment with naked dsRNA, as was shown for the *FgVe1* gene. At 7 dpi, the observed picture changed dramatically. In samples treated with naked FgVe1_540 dsRNA, non-significant 1.51-fold up-regulation of *FgVe1* expression was observed (*p* = 0.147). *Tri5* and *Tri6* genes also demonstrated slight up-regulation (2.03- (*p* = 0.048) and 1.38- (*p* = 0.1097) fold, respectively). In contrast, all the tested genes remained silenced at 7 dpi under treatment with the CaPs:FgVe1_540 dsRNA complex, showing significant down-regulation (11.46-fold (*p* = 0.00376) for *FgVe1*, 2.7-fold (*p* = 0.00413) for *Tri5*, 3.22-fold (*p* = 0.038) for *Tri6*, 2.43-fold (*p* = 0.0222) for *Pks12* and 2.4-fold (*p* = 0.042) for *AurR1*). Interestingly, it was observed that samples treated by either naked FgVe1_540 dsRNA or CaPs:FgVe1_540 dsRNA differed from the control samples by mycelium color (see Figure S4, photographs were taken at 7 dpi), and this fact can be related to the inhibition of *Pks12* and *AurR1* gene expression. At 10 dpi, the *FgVe1* gene was up-regulated (1.96-fold, *p* = 0.02) under naked dsRNA treatment and non-significantly down-regulated (1.29-fold, *p* = 0.594) when treated with CaPs:dsRNA nanocomplex. In samples, treated with naked FgVe1_540 dsRNA, statistically significant up-regulation of *Tri5* gene expression was observed (1.91-fold, *p* = 0.022), whereas in samples treated with CaPs:dsRNA nanocomplex, there was almost no difference in the control (1.02-fold change (f.c.) up-regulation, *p* = 0.973). Expression of the *Tri6* gene was insignificantly up-regulated under both treatments (1.52-fold (*p* = 0.143) with naked dsRNA and 1.24-fold (*p* = 0.221) with the nanocomplex). *Pks12* gene was up-regulated (1.5-fold, *p* = 0.092) under naked dsRNA and slightly down-regulated under the nanocomplex treatment. Relative expression of the *AurR1* gene was at the level of the control sample in cultures treated with naked FgVe1_540 dsRNA and was down-regulated (1.54-fold, *p* = 0.065) in cultures treated with the CaPs:FgVe1_540 dsRNA complex. Interestingly, a slight inhibition of expression of the *Pks12* and *AurR1* genes was observed in control samples containing CaPs, including CaPs suspension and CaPs:ChS dsRNA nanocomplexes, although this down-regulation was statistically non-significant.

### 2.4. Effect of dsRNA Treatment on DON Biosynthesis by F. graminearum

**Figure 5:**
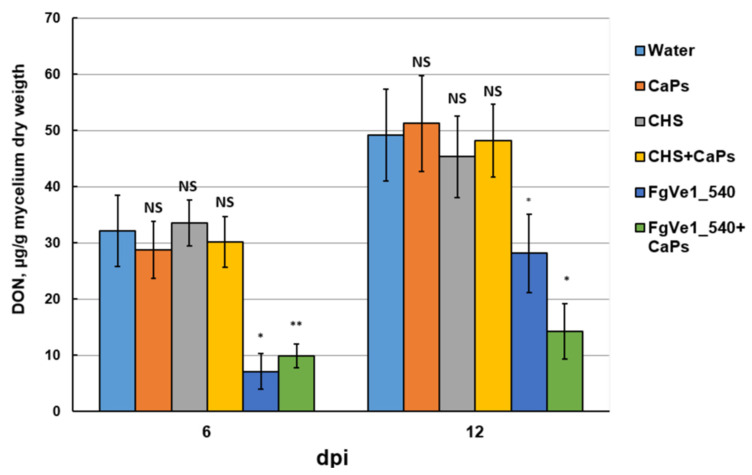
Effect of FgVe1_540 dsRNA and CaPs:FgVe1_540 dsRNA nanocomplexes treatment on DON accumulation in liquid cultures of *F. graminearum* MFG 58918. DON content is expressed as µg per g of mycelium dry weight. Bars represent mean ± SD (n = 4). Symbols indicate the significance levels of differences according to the Tukey HSD test: ** *p* < 0.01; * *p* < 0.05; NS—no significant difference.

Since dsRNA treatment led to the silencing of trichothecene biosynthetic genes, its influence on DON accumulation in liquid media was also assessed. Analyses were performed at 6 and 12 dpi. At 6 dpi, DON content was decreased by approximately 78% in samples treated with naked FgVe1_540 dsRNA and by approximately 70% in samples treated with CaPs:FgVe1_540 dsRNA nanocomplexes (Figure 5). By 12 dpi, a fourfold increase in DON concentration was observed in cultures containing naked specific dsRNA, and this result correlates well with the restoration of *Tri5* and *Tri6* gene expression, observed by 7 dpi and later. In contrast, CaPs:dsRNA-treated samples contained almost two times less DON at 12 dpi, indicating the prolonged action of the nanocomplex.

### 2.5. Spraying Detached Leaves with Naked dsRNA and CaPs:dsRNA Nanocomplexes and Evaluation of Their Effect on F. graminearum Growth

**Figure 6:**
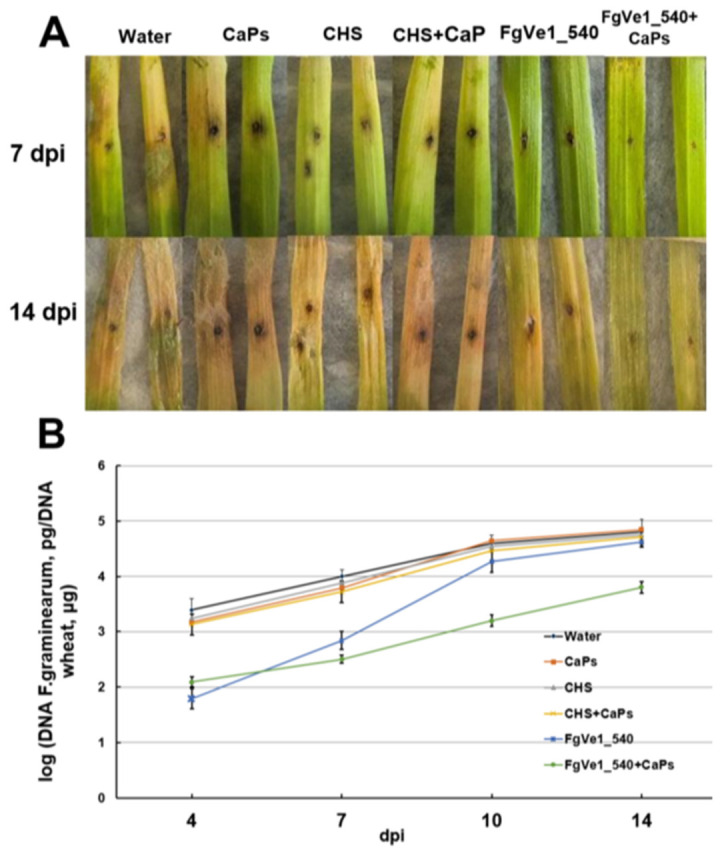
Effect of FgVe1_540 dsRNA and CaPs:FgVe1_540 dsRNA nanocomplexes treatment on *F. graminearum* MFG 58918 growth on detached wheat leaves. Photographs were taken at 7 and 14 dpi (**A**). Dynamics of *F. graminearum* MFG 58918 growth in treated and control detached wheat leaves expressed as log10 (fungal DNA, pg/wheat DNA, µg). Analyses were carried out at 4, 7, 10, and 14 dpi (**B**).

To evaluate the effect of dsRNA on fungal development in plant tissues, we carried out a visual assessment of detached wheat leaves sprayed with FgVe1_540 dsRNA, CaPs:FgVe1_540 dsRNA nanocomplexes, and control solutions. At 7 dpi, disease symptoms, expressed as black necrotic points and bleaching, were significantly more pronounced on control leaves than on leaves treated with either naked specific dsRNA or CaPs:dsRNA nanocomplexes (Figure 6A). By 14 dpi, aerial mycelium was observed on control leaves and absent on treated ones (mycelium can be seen at the upper parts of the leaves, Figure 6A). To estimate fungal growth more precisely, we analyzed the fungal DNA content in leaf tissue by qPCR using *F. graminearum*-specific primers and a hydrolysis probe. The limit of detection was around 10 pg of fungal DNA per reaction (Figure S5). The relative quantity of specific DNA was estimated as the ratio between fungal and plant DNA (logarithmic, see Figure 6B). Measurements were carried out at 4, 7, 10, and 14 dpi. It was shown that fungal growth was significantly inhibited during the early stages of infection (4 dpi). This fact was expressed as more than a tenfold difference between fungal DNA content in leaves treated with specific dsRNA or CaPs:dsRNA nanocomplexes and control (untreated) leaves. On the leaves treated with naked dsRNA, active fungal growth started after 7 dpi, and by 14 dpi, the fungal DNA content was almost equal to that in control leaves. At the same time, *F. graminearum* grew slowly on the leaves treated with CaPs:FgVe1_540 dsRNA nanocomplexes, and an approximately tenfold difference in DNA content was maintained during the entire period of observation.

### 2.6. Effect of Spraying Detached Leaves with Naked dsRNA and CaPs:dsRNA Nanocomplexes on F. graminearum Gene Expression

**Figure 7:**
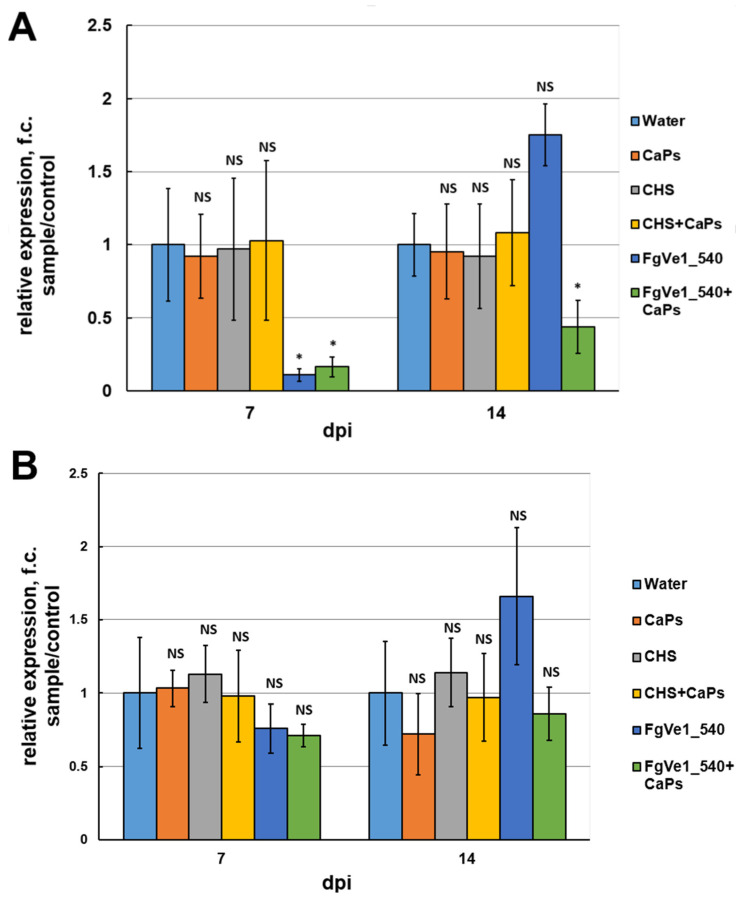
Effect of FgVe1_540 dsRNA (75 ng/μL) and CaPs:FgVe1_540 dsRNA nanocomplexes (375:75 ng/μL) treatment on the relative expression of the *FgVe1* (**A**) and *Tri5* (**B**) genes in detached wheat leaves. Analyses were carried out at 7 and 14 dpi. Control treatments included (i) sterile water (expression levels of target genes in these samples were considered as 1.0); (ii) CaPs suspension (375 ng/μL); (iii) ChS dsRNA (75 ng/μL); (iv) CaPs:ChS dsRNA nanocomplex (375: 75 ng/μL). The experiment was carried out in three biological replicates. The *FgVe1* expression level in the control (untreated) leaves was considered as 1.0. The *Tef1α* gene was used as a reference. Bars represent mean ± SD (n = 3). Symbols indicate the significance levels of differences according to the Tukey HSD test: * *p* < 0.05; NS—no significant difference.

To assess the silencing effect of FgVe1_540 dsRNA and CaPs:FgVe1_540 dsRNA nanocomplexes on gene expression of *F. graminearum* during infection on detached leaves, analyses of relative expression of *FgVe1* and *Tri5* genes were carried out at 7 and 14 dpi. At 7 dpi, the *FgVe1* expression was 9.01-fold (*p* = 0.016) down-regulated in leaves treated with naked dsRNA and 6.03-fold (*p* = 0.02) down-regulated in leaves treated with CaPs:dsRNA nanocomplex (Figure 7A). At the same time, the *Tri5* gene was down-regulated only slightly and statistically non-significantly (1.32-fold, *p* = 0.212) in leaves treated with naked dsRNA and 1.41-fold (*p* = 0.452) down-regulated in nanocomplex-treated leaves (Figure 7B). By 14 dpi, the *FgVe1* gene enhanced its transcription level and was up-regulated (1.75-fold, *p* = 0.072) in the leaves sprayed by naked FgVe1_540 dsRNA solution. In contrast, in the leaves sprayed by the nanocomplex, *FgVe1* expression remained partially inhibited (2.26-fold down-regulation, *p* = 0.0124). The *Tri5* gene was slightly up-regulated in naked dsRNA-sprayed leaves (1.66-fold, *p* = 0.083) and slightly down-regulated (1.16-fold, *p* = 0.112) in the leaves treated with the CaPs:dsRNA nanocomplex (Figure 7B).

## 3. Discussion

*Fusarium* species and their mycotoxins are among the most serious threats to agriculture and the food industry. Currently, fungicides remain the most applied technique, but limitations conditioned by safety concerns and resistance development have led to the demand for searching for novel approaches to manage the pathogens. Spraying plants with dsRNAs or siRNAs proved effective to combat different plant pathogens, including viruses, bacteria, nematodes, and fungi [57,58,59,60]. Koch et al., 2013 [61], first demonstrated that treatment of *F. graminearum* axenic cultures with dsRNA, complementary to the partial sequences of three *CYP* genes responsible for ergosterol biosynthesis, resulted in inhibition of fungal growth and altered morphology. Later, these authors showed that spray-induced silencing using the same CYP3-targeting dsRNA protects not only directly sprayed but also distant parts of detached barley leaves, as well as the fact that the plant vascular system is involved in dsRNA movement through the plant [26]. These data provided the basis for further development of SIGS-based assays against fusariotoxin producers. In the last few years, the number of publications devoted to the application of RNAi-based techniques to control *Fusarium* spp. and other plant-pathogenic fungi has increased dramatically, but some issues are still to be addressed before this approach becomes massively used in practice.

In this study, the *FgVe1* gene, related to a wide range of processes in *F. graminearum*, was selected as a target for silencing. Previously, homologs of this gene were successfully used for host-induced silencing to control wilt of banana, caused by *F. oxysporum* f. sp. *cubense* [62], and aflatoxin-producing *A. flavus* in groundnut [63]. Our suggestion was that silencing of the *FgVe1* gene could result in inhibiting expression of other genes, including those related to growth, virulence, and trichothecene biosynthesis. Since trichothecenes are a fungal virulence factor themselves, a reduction in the biosynthesis of these compounds can be an additional factor of plant protection.

It is well-known that dsRNAs of different sequences, targeting the same gene, can demonstrate different silencing efficiency when applied in vitro or in planta. For instance, Song et al., 2018 [64], showed that eight fragments of the *Myo5* gene of approximately equal lengths differed significantly in terms of gene silencing efficiencies and corresponding phenotypic effects. Similar results were obtained by Gu et al., 2019 [65], for dsRNAs derived from the *β*-*tub* gene of *F. asiaticum*. We compared three dsRNAs (150, 370, and 540 bp long) at different concentrations by their ability to silence the *FgVe1* gene expression in liquid cultures of *F. graminearum* MFG 58918. Six concentrations ranging from 20 to 1000 ng/mL of media were tested. Concentrations of 20 and 50 ng/mL were inefficient regardless of which dsRNA was used. The greatest effect was achieved by applying the longest variant (FgVe1_540 dsRNA), which was able to induce silencing at a concentration range from 100 to 1000 ng/mL. When applied at 500 ng/mL, FgVe1_540 dsRNA induced 19.78-fold down-regulation of *FgVe1* expression. Such a concentration is quite standard and similar to those used in some previous studies [32,50,52,66]. Interestingly, the highest concentration of FgVe1_540 dsRNA (1000 ng/mL) was less efficient (10.92-fold down-regulation). The fact that higher concentration is not more efficient can possibly be explained by the overloading of fungal RNAi machinery with dsRNA at certain concentrations and its inability to process all the dsRNA simultaneously. A surprising result of our study is that the most GC-rich tested dsRNA (FgVe1_370, GC = 57.3%) was the least efficient, despite it being commonly believed that regions with higher GC content are preferable for Dicer [67,68].

Further, it was demonstrated that silencing of the *FgVe1* gene, as expected, resulted in inhibition of the expression of genes related to fungal secondary metabolism, such as *Tri5* and *Tri6*, which are necessary for trichothecene biosynthesis, as well as *Pks12* and *AurR1*, participating in the biosynthetic pathway for production of aurofusarin. Down-regulation of the expression of *Tri5* and *Tri6* genes led to a reduction in DON biosynthesis. This result is of high practical value because mycotoxin biosynthesis inhibition is probably a more important task than inhibiting fungal growth, as mycotoxins are highly toxic to humans and play a significant role in pathogenic processes in plants.

Among the main challenges limiting the field application of SIGS is the fact that dsRNA can be degraded rapidly in the environment, in particular due to nucleases, as well as the inability of several fungi to maintain silencing. According to Song et al., 2018 [64], dsRNA remains stable on the plant surface for up to 8 days. The authors also concluded that in the liquid culture of *F. asiaticum*, a member of the *F. graminearum* species complex, the silencing effect is limited to 7 days since this species is unable to produce secondary siRNAs. One way of addressing this problem is by using nanocarriers, protecting dsRNA from degradation and providing its targeted delivery to a pathogen. Recently, several types of nanoparticles have been used in agriculture to improve dsRNA effects against plant pathogens, including fungi. For instance, LDH were applied to the topical delivery of dsRNA, protecting tomato plants from crown and root rot, caused by *F. oxysporum* f. sp. *radices-lycopersici* [32]. Recently, LDH were successfully used to improve SIGS efficiency in the management of *Botrytis cinerea* on lettuce: it was demonstrated that LDH:dsRNA nanocomplexes provided prolonged protection up to 27 days in comparison to naked dsRNA [34]. Wang et al. [33] showed that chitosan nanoparticles improve the stability of dsRNA and enhance the efficiency of RNAi-based protection against *Rhizoctonia solani*. Therefore, the use of nanocarriers helps to overcome some limitations of RNAi-based approaches and opens up prospects for their further development. In our study, the carriers chosen were calcium phosphate nanoparticles. Calcium phosphate is a biodegradable and biocompatible material, which has been widely applied for scientific and practical purposes. In plant biotechnology, CaP nanoparticles were successfully used as carriers of the pCambia1301 plasmid for the transformation of brown mustard (*Brassica juncea* L.) plants [69]. According to the results, the CaPs-based transformation efficiency was 80.7%, which is significantly higher than the efficiency of *Agrobacterium tumefaciens*-mediated transformation (54.4%). CaPs were also applied for plant protection. For instance, CaPs proved effective nanocarriers to deliver an elicitor methyl jasmonate, improving the protective properties of grapes and enhancing wine quality [70]. Calcium phosphate polymorph hydroxyapatite has been applied to increase tomato growth parameters and to control the *Meloidogyne incognita* nematode [71]. Hydroxyapatite served as a component of a nanocarrier complex, encapsulating bioactive extract from cat’s claw (*Uncaria tomentosa*) for post-harvest protection of different fruits against fungal pathogens [72]. In medicine, CaPs have been used for a targeted delivery of siRNA in the treatment of inflammatory disorders of the lung [73] and tumor therapy [74,75]. However, there is no data on using CaPs as siRNAs or dsRNAs carriers for plant pathogens management. In a previous work [76], stable CaPs were synthesized in the presence of sodium citrate as a stabilizing agent without cooling.

In this study, we employed spherical CaPs consisting of hydroxyapatite and amorphous calcium phosphate, which formed complexes with RNA at a 5:1 weight ratio in the presence 5 mM Ca^2+^ buffer. The incorporation of dsRNA into the CaPs resulted in a doubling of their height, as measured by AFM, increasing from 35 nm to 72 ± 10 nm. Morphological analysis revealed that the resulting complexes were spherical and possessed an increased surface roughness.

Our suggestion was that CaPs complexed with dsRNA could enhance and prolong their action. To verify this hypothesis, we used CaPs:FgVe1_540 dsRNA nanocomplexes for the treatment of liquid cultures of *F. graminearum* and infected detached wheat leaves, and compared the effects of nanocomplexes and naked dsRNA. Different types of controls were used, including CaPs suspensions, non-specific dsRNA complementary to the *ChS* gene of hop, and CaPs:ChS dsRNA nanocomplexes. The key parameters to estimate were efficiency and duration of target gene silencing, inhibition of DON accumulation, and influence on fungal growth on detached leaves. It was established that neither CaPs themselves nor non-specific dsRNA affected fungal growth and target gene expression. An intriguing result of this study was that naked dsRNA and dsRNA complexed with CaPs differ in their silencing efficiencies at different time points. At the early stages of fungal growth (4 dpi in liquid media and 7 dpi in detached leaves), naked dsRNA caused stronger down-regulation of *FgVe1* expression than dsRNA complexed to CaPs (18.54-fold vs. 11.1-fold in liquid culture and 9.01-fold vs. 6.03-fold in detached leaves, respectively). A possible explanation of this fact is that the availability of dsRNA is one of the crucial factors determining silencing efficiency. In the early stages of growth, naked dsRNA presents in large excess and can easily be processed by fungus, whereas in nanocomplex dsRNA is linked and not so available. Mosa and Youseff, 2021 [32], described the silencing dynamics at 0, 24, 48, 72, and 96 h after dsRNA treatment of *F. oxysporum*. It was shown that the most significant reduction in target transcript levels was observed in the period from 48 to 96 h. We can suggest that after this time, dsRNA begins to be exhausted and degraded by cellular enzymes. Thus, silencing diminishes, and this is especially a problem for organisms unable to produce secondary siRNAs. Loading dsRNA into nanoparticles provides protection of the molecule from environmental degradation and its prolonged release and supply into culture or plant surface. We demonstrated that in samples treated with CaPs:dsRNA nanocomplexes, *FgVe1* remained silenced at 7 and even 10 dpi in liquid culture, whereas in samples treated with naked dsRNA, it was slightly up-regulated. The same picture was observed for the genes of trichothecene biosynthesis, especially *Tri5*, which was silenced under naked dsRNA at 4 dpi and two-fold up-regulated at 7 dpi. This up-regulation can be explained by the fact that genes related to growth and secondary metabolism are often actively expressed in the early stages of fungal growth, and this expression decreases in 7–10 days. In turn, in samples treated with naked dsRNA, fungus actively expresses these genes after dsRNA is exhausted, and this results in observed up-regulation compared to the control.

We also demonstrated that the application of CaPs:dsRNA nanocomplexes kept DON biosynthesis partially inhibited at 12 dpi. Similarly to gene expression silencing, naked dsRNA was more efficient at an earlier stage (6 dpi), but later DON accumulation was restored. Moreover, we observed the change in color of fungal cultures treated with both naked dsRNA and CaPs:dsRNA nanocomplexes. Application of CaPs:FgVe1_540 dsRNA nanocomplexes on detached wheat leaves resulted in prolonged down-regulation of the *FgVe1* gene up to 14 dpi, as well as inhibition of fungal growth determined by DNA accumulation. At the same time, inhibition of *Tri5* gene expression was relatively weak at both 7 and 14 dpi. This result looks strange considering that the *Tri5* gene is under transcriptional control of *FgVe1* and its silencing was demonstrated in liquid media. However, in plant tissues, the genes of a trichothecene biosynthetic cluster can be weakly expressed if the fungus exploits other pathogenicity mechanisms. Moreover, the highest expression of the *Tri5* gene is usually observed in the first days of infection and could be decreased by 7 dpi, and therefore, there was no significant difference between treated and control leaves. On the other hand, up-regulation of *Tri5* expression was observed after the termination of the naked dsRNA effect by 14 dpi. Probably, the fungus uses trichothecene biosynthesis to compensate for the growth inhibition under the action of dsRNA.

Therefore, in this study, we demonstrated the principal possibility of using CaPs forming complexes with dsRNA as carriers, which prolongs the silencing effect in comparison to a naked molecule. These results provide important information for the further development of RNAi as a tool for the management of toxigenic fungi. At the same time, the final goal of this research, undoubtedly, should be the development of dsRNA-based biopesticides for plant protection, which will require further studies, including testing on whole plants and field trials under different environmental conditions.

## 4. Materials and Methods

### 4.1. Fungal and Plant Material, Growth Conditions

DON-producing *F. graminearum* strain MFG 58918 was isolated from wheat in the Krasnodar region in 2016 and provided by All-Russian Institute of Plant Protection (St. Petersburg-Pushkin, Russia). It was maintained on Potato Dextrose agar (PDA) plates at 25 °C. Conidial suspensions of *F. graminearum* MFG 58918 were obtained from 7-day-old mycelium by washing off with sterile water, followed by filtering through sterile cotton wool. The concentrations of conidial suspensions were measured using a hemocytometer. To induce mycotoxin biosynthesis, Myro medium [77] was used; 5 mL flasks were incubated at 25 °C and 250 rpm in Biosan Environmental Shaker-Incubator ES-20 (Biosan, Riga, Latvia) for 12 days in the dark.

The susceptible spring wheat (*Triticum aestivum* L.) cultivar Daria was used to perform the detached leaves assay. Plants were grown in a greenhouse at 60% relative humidity and temperature 25 °C (day, 16 h) and 22 °C (night, 8 h) for 20 days. After inoculation and dsRNA treatment, the detached leaves were incubated in Petri dishes under the same conditions for 14 days.

### 4.2. Nucleic Acids Extraction

RNA from fungal biomass grown in liquid cultures and detached wheat leaves was extracted using RNeasy Plant Mini Kit (Qiagen, Hilden, Germany) according to the manufacturer’s instructions. Before extraction, samples were lyophilized in VirTis BenchTop 2K XL freeze drier (SP Scientific, Stone Ridge, NY, USA) for 1.5 h and ground in liquid nitrogen.

DNA from fungal mycelium and detached wheat leaves was extracted using PROBA-TsTAB commercial kit (DNA-technology, Moscow, Russia) following the manufacturer’s protocol.

Concentration and quality of the extracted samples were measured using a Qubit 3.0 fluorometer (Life Technologies, Waltham, MA, USA) and NanoVue spectrophotometer (GE HealthCare, Chicago, IL, USA), respectively.

### 4.3. Primer Design

Primers for dsRNA synthesis were designed based on *F. graminearum* PH-1 *FgVe1* gene sequence (accession number XM_011318944). In total, three different fragments (148, 370, and 540 bp, see Figure S1) were selected. To synthesize non-specific (control) dsRNA, primers for amplifying a fragment of chalcone synthase gene (*ChS*) of hop (accession number NM_001427915, see Figure S2) were designed. All the primers used in the study are listed in Table 1. Physical and chemical properties of primers were analyzed using Oligo 6.0 software. Primer specificity was verified by BLAST (https://blast.ncbi.nlm.nih.gov/Blast.cgi (accessed on 30 March 2025)). Potential off-targets were predicted using SiFi21 software (https://sourceforge.net/projects/sifi21 (accessed on 5 May 2025)).

### 4.4. In Vitro Transcription

Amplified fragments of *FgVe1* and *ChS* genes were cloned into pAL2-T plasmid vector (Evrogen, Moscow, Russia) and used as templates for dsRNA synthesis. In vitro transcription was carried out using HiScribe^®^ T7 High Yield RNA synthesis kit (New England Biolabs, Ipswich, MA, USA) according to the manufacturer’s instructions. After synthesis, dsRNAs were treated with DNAse E (Evrogen, Moscow, Russia) according to the manufacturer’s instructions, and purified using phenol and chloroform.

### 4.5. CaPs Preparation and CaPs:dsRNA Nanocomplexes Formation

CaP nanoparticles were synthesized as described earlier [76]. The method without cooling was used. Briefly, we mixed 12.5 mM potassium phosphate and 15.6 mM sodium citrate aqueous solutions (5:1 *V/V*) and adjusted the pH of the mixture to 8.8. Then, 200 W ultrasonic treatment using a Sonopuls ultrasonic homogenizer (Bandelin, Berlin, Germany) for 20 min was performed. At the start of ultrasonic treatment, 12.5 mM calcium chloride solution (5 vol.) was added to the mixture. Before the synthesis, all the prepared solutions were filtered through 0.45 µm syringe filters (Merck Millipore, Darmstadt, Germany).

The concentration of the obtained CaPs in the suspension was 1 mg/mL. The mean hydrodynamic diameter of CaPs by DLS and ζ-potential were measured using a Zetasizer Nano ZS (Malvern Co., Ltd., Malvern, UK).

The morphology and shape of CaPs were studied using scanning transmission electron microscopy (STEM) using scanning electron microscope Hitachi S5500 (Hitachi High-Technologies Corporation, Tokyo, Japan). A 3 µL aliquot of the CaPs suspension, pre-dialyzed against deionized water, was deposited onto a 3 mm copper grid coated with formvar and carbon. The sample was air-dried and subsequently vacuum-desiccated at 10^−5^ Torr for 3 h. Prior to imaging, the grid was transferred to the microscope chamber and held at 10^−5^ Torr for 12 h. Imaging was performed using a scanning electron microscope operated at 30 kV in STEM mode, utilizing both transmission (BF-STEM) and reflection (SE) mode detectors.

The phase composition was characterized using a Rigaku Miniflex 600 X-ray diffractometer (Rigaku, Tokyo, Japan) with Cu Kα radiation and a Ni-Kβ filter. Prior to analysis, the CaPs suspension in deionized water was lyophilized, and the resulting powder was resuspended in ethanol.

CaPs:dsRNA nanocomplexes were prepared by mixing a CaP nanoparticle suspension and dsRNA solution (5:1 *w/w* ratio) in the presence of 5 mM CaCl_2_ followed by incubation on ice for 60 min.

The morphology of CaPs and CaPs:dsRNA complexes was studied using atomic force microscopy (AFM) with a FemtoScan atomic force microscope (ATC, Moscow, Russia). AFM scanning was carried out in the air in a resonant mode with an NSG10 cantilever on freshly cleaved graphite (highly oriented pyrolytic graphite) and mica substrates. The sorption of dsRNA on CaPs was carried out in the presence of Ca^2+^. We used diluted samples of CaPs (0.05 μg/μL), as well as samples of CaPs:dsRNA complexes. The results were processed using FemtoScan Online software, version 2.3.239 [78].

### 4.6. dsRNA Treatment of Liquid Culture Media

To compare silencing efficiencies and select an optimal concentration, three specific dsRNAs were diluted in 200 µL of sterile water and added to 15 mL Falcon tubes containing 5 mL of Myro media (final dsRNA concentrations 20, 50, 100, 250, 500, and 1000 ng/mL of media). After 24 h, the media were inoculated with 200 µL of *F. graminearum* conidial suspensions (1 × 10^5^ conidia/mL). RNA extraction was carried out at 4 dpi.

After the most efficient dsRNA and its optimal concentration were determined, the following treatments of *F. graminearum* liquid cultures were performed: (i) 200 μL of sterile water; (ii) 200 μL of CaPs suspension (62.5 ng/μL); (iii) 200 μL of ChS dsRNA (12.5 ng/μL); (iv) 200 μL of CaPs:ChS dsRNA nanocomplex (62.5:12.5 ng/μL); (v) 200 μL of FgVe1_540 dsRNA (12.5 ng/μL); (vi) 200 μL of CaPs:FgVe1_540 dsRNA nanocomplex (62.5:12.5 ng/μL). RNA extractions for relative gene expression analysis were carried out at 4, 7, and 10 dpi. Samplings for mycotoxin analysis were carried out at 6 and 12 dpi.

The experiments were carried out in four biological replicates with two technical replications for each sample.

### 4.7. Detached Leaf Assay

Detached leaves of 20-day-old wheat were sterilized with 1% sodium hypochlorite for 30 s and washed in sterile water for 1 min. After that, the leaves were placed on Petri dishes containing moistened filter paper. Upper and lower parts of the leaves were covered with cotton disks, soaked in Murashige and Skoog (MS) medium [79]. Before spraying, the leaves were wounded by a sterile needle. For spray application, both FgVe1_540 and non-specific ChS dsRNAs were diluted in 200 µL of sterile water to a final concentration of 75 ng/µL (approximately 15 μg per plate containing six leaves). The same volume of CaPs:dsRNA nanocomplexes and CaP suspension (375 ng/µL) was prepared; 200 µL of sterile water was used as a control. Leaves were sprayed using a 5 mL spray flask. After being sprayed, the leaves were dried for 1 h; 24 h later, they were drop-inoculated with 5 µL of *F. graminearum* conidial suspension (1 × 10^5^ conidia/mL). Visual assessments and RNA extractions for relative gene expression analysis were carried out at 7 and 14 dpi. DNA extractions for quantification of fungal biomass were carried out at 4, 7, 10, and 14 dpi. Experiments were carried out in three biological replicates with two technical replicates (qPCR).

### 4.8. qPCR Experiments

cDNA was synthesized using MMLV RT kit (Evrogen, Moscow, Russia) with a 10-mer random primer according to the manufacturer’s instructions. The qPCR reactions were carried out using primer sets listed in Table S1. All the oligonucleotides except the ones for the *AurR1* gene were developed earlier [77]. The *Tef1α* gene was used as a reference. The relative expression of the genes of interest was estimated by the 2^−ΔΔCt^ method [80] using QGene software, version 4.4.0. The results were expressed as fold changes (f.c.) in transcript level of a corresponding gene between the treated sample and the control (taken as 1.0).

Quantification of fungal DNA was carried out by qPCR using a primer pair (F: 5′-ACTCGAGCGACAGGCGYC-3′; R: 5′-TTCCTATTGACAGGTGGTTAGTGA-3′) and a TaqMan^®^ fluorogenic probe (5′-BHQ1-CCATTCCCTGGGCACTCA(FAMdT)CATCACGTGTC-3′) complementary to the *Tef1α* gene sequences and strictly specific to *F. graminearum*. Wheat DNA quantity was determined using the following primer pair: 5′-TCGGAGATAAGCCAGGTGGAC-3′ (F), 5′-TGCTGACATACTGGAACATCTCG-3′ (R) and probe: 5′-BHQ1-TAACATCCATTG(FAMdT)CAGCTATAGCCGAGCATCT-3′ complementary to the wheat *Ef1α* gene. Sensitivity of both the assays was evaluated by standard curves generated through qPCR of ten-fold dilutions of pure DNA samples (10^1^–10^7^ per reaction, Figure S5A,B). The results were represented as Log_10_ (pg of *F. graminearum* DNA/μg of wheat DNA).

All the qPCR reactions were performed in a DT-96 detecting thermocycler (DNA-technology, Moscow, Russia) according to the following protocol: 94 °C for 1 min (1 cycle); 94 °C for 10 s, 64 °C for 30 s, 72 °C for 5 s (35 cycles); 72 °C for 2 min. The reaction mix components were described earlier [81].

### 4.9. DON Content Analysis

A fungal culture medium was separated from the mycelium biomass by centrifugation at 7000× *g* for 10 min and additionally filtered through a paper filter (Whatmann, Clifton, NJ, USA). Concentrating was performed with a Manifold 12-valve medium pressure system (Macherey-Nagel, Düren, Germany) using Strata C18E cartridge columns (55 µm, 70 Å) (Phenomenex, Torrance, CA, USA). After the release of all unbound components in 0.1% trifluoroacetic acid, elution of the mycotoxin-containing fraction was carried out with 60% aqueous acetonitrile supplemented with 0.1% trifluoroacetic acid; the resulting eluate was sub-evaporated using a SpeedVac vacuum centrifuge (Labconco, Kansas City, MO, USA). Separation of mixture components and detection of DON was performed using an AGILENT 1200 Series HPLC system (Agilent Technologies, Santa Clara, CA, USA) supplemented with UV/VIS detector and automated Autosampler for analytical reversed-phase HPLC in a linear gradient of buffer B (80% acetonitrile supplemented with 0.1% trifluoroacetic acid) versus buffer A (0.1% trifluoroacetic acid): 5–40% B for 30 min, 40–75% B for 15 min, 75–90% B for 5 min, isocratic elution of 90% B for 5 min. An XBRIDGE BEH C18 4.6 × 250 mm, 5 μm, 130 Å column (Waters, Wexford, Ireland) was used at a flow rate of 0.95 mL/min and absorbance at UV wavelength of 254 nm. Identification of DON was performed by comparing its retention times relative to analytical standard M0101QN (Evrika, Moscow, Russia) previously applied to the indicated column under the same conditions at a concentration of 5 µg/mL.

### 4.10. Statistical Analysis

Statistical data analysis was performed using one-way ANOVA and with Tukey’s test as a post hoc test for multiple comparisons. Differences between samples with *p* > 0.05 were considered as non-significant.

## 5. Conclusions

There is an ongoing need to combat mycotoxigenic fungi affecting agricultural plants using highly efficient and environmentally safe approaches. RNAi-based methods, including SIGS, have been considered as a possible replacement for traditional fungicides, but some issues should be addressed before it is possible. These issues include the development of cheap and simple methods of dsRNA production, selecting the most appropriate target genes, better understanding of uptake mechanisms, and overcoming problems, conditioned by the instability of dsRNA in laboratory and field conditions. The use of nanocarriers is a possible way to protect dsRNA from environmental degradation and improve its effect. In this work, we showed that CaPs represent a prospective biocompatible nanocarrier for dsRNA to be used in plant protection against toxigenic *F. graminearum*. These compounds are relatively cheap, easy to synthesize, and simple to aggregate with dsRNA. In this work, we demonstrated that CaPs enhanced the silencing effect caused by dsRNA and prolonged its action. In addition, the presence of CaPs in fungal culture did not alter fungal growth and had no significant effect on the expression of fungal genes. Therefore, the obtained results look promising in terms of developing improved RNAi-based methods to control mycotoxigenic fungi. However, it is definitely a basis for further studies, including large-scale field trials, to assess the possibility of using this approach in practice.

## Figures and Tables

**Table 1:** List of primers used for dsRNA synthesis. Sequences complementary to *FgVe1* and *ChS* genes are indicated in bold.

Target Gene	Primer Names	Sequence, 5′-3′	dsRNA Length, bp
*FgVe1*	FgVe1_150F	TAATACGACTCACTATAGGG**ATGGCGACACCTTCAGCAA**	148
	FgVe1_150R	TAATACGACTCACTATAGGG**TCATACCAGAACCGCATGCTC**	
*FgVe1*	FgVe1_370F	TAATACGACTCACTATAGGG**AGCGTCGCGAGGAAGATTTC**	370
	FgVe1_370R	TAATACGACTCACTATAGGG**GGACTGTAACCACGGTGAGACC**	
*FgVe1*	FgVe1_540F	TAATACGACTCACTATAGGG**GCTAACAGCGACCGCCGA**	540
	FgVe1_540R	TAATACGACTCACTATAGGG**GGTGTCGCGCTTCCTCATG**	
*ChS*	CHS_315F	TAATACGACTCACTATAGGG**ATGCACTTAACTGAGGAGATCCT**	315
	CHS_315R	TAATACGACTCACTATAGGG**TGGCGACTCTGAGGACCGT**	

## Data Availability

All data generated or analyzed during this study are included in this publication.

## Supplementary Materials

The following supporting information can be downloaded at: https://www.mdpi.com/article/10.3390/ijms262010021/s1.
