# Supplementary material for: CaP Nanoparticles Improve the Effect of dsRNA on Gene Expression, Growth, and Mycotoxin Production of Toxigenic Fusarium graminearum
Source: Int J Mol Sci. 2025 Oct 15;26(20):10021. doi: 10.3390/ijms262010021 (PMC12562286; doi:10.3390/ijms262010021)
Supplement: Supplementary file 1 [file ijms-26-10021-s001.zip › ijms-3884537-supplementary.pdf]

ATGGCGACACCTTCAGCAATTCCCACAAACTCAAAACGCGACACCACCGCTCAGTTTCACAGAGTCACGA
GAGGAAACCGGAGCCTCTGGTACCAGCTGACTGTCCTGCAACAACCAGAGCGAGCCCGAGCATGCGGTTC
TGGTATGAAAGCTAACAGCGACCGCCGACCCGTTGACCCTCCGCCTGTTGTCGAGCTACGAATTGTTGAA
GGCCCCACTCTTGAAGAGGGCAAGGATGTAACATTTGATTATAACGCCAACTTCTTTCTCTATGCCAGTC
TCGAGCATGCTCGCCCAATAGCCTCTTGCCGTGTTTCAACGCCCACCACCAACAACCCGCCTATTCTTAC
TGGTGTTCCCGCCTCGGGCATGGCCTATCTTGACCGACCATCCGAAGCCGGCTACTTTATCTTCCCCGAT
CTTTCTGTGCGCCATGAGGGCCTCTACCGTCTGACTTTCAGCTTATTCGAGACTACCAAGGAGGAGCAGG
ATTTCGACATTCAGCCTGCCGATGGCGATCTTCCACCTGGCGTGGACTTCCGAATGGAGATTAAGACCGA
CCCATTCTCAGTTTTCAGCGCCAAGAAGTTCCCCGGTCTCATGGAGAGCACGCAGCTCAGCAAGACAGTC
GCTGACCAGGGCTGCCGAGTTCGAATTCGTCGTGATGTTCGCATGAGGAAGCGCGACACCAAGTCAGGTG
GCAACAACAATAACAACAACAACGCTGGTAACAATGCTGGAAACAACGGCTTTGAGCGTCGCGAGGAAGA
TTTCGGTCGTAGAAGGACTTCCACCAGCACCTACGTTCCACCCTCACCTTCGGTCTACTCCACGGAGGGC
CACTACCGTCGAGACTCGCAAGCGTCATACCCTCCCACGCCTGCTGCTGCTCCTCTTCCTCGCATGAACA
CTGAGCCATCTCGCGGATCTATCAAGATTTCTGCTCTAGTCGAGCCCATGCCAGTCATTGAGCCTCAGGT
CGACCCTCTCCCTGAACTTCCCCCCGTCAATGTTGGCGGTAAGCGCAAACACGAGAGCGTCTTTGCCCAA
AACACCAGGCCACTTTTCAACGGTCAAAGACAAATGGACCCTCACTACGGCCGGTCTCACCGTGGTTACA
GTCCCGACCACGACCAAGGCTCTTACTCTCGAGCTGATGGTCAGATCAGCGTTATTCAATTCAACAAATA
CGAGTATTAAACATCGACATTCTTACTTCTTGACTAGAAAGACATACCGGCGTTAGAATGTTGTGTATTC
CTATCTACAGGAGGAGGACGGTAAGATGATGGAAGATACATGAGCGACAATGTTTAGGAGTTCAGGCGCA
ATTCATGTGGATCAAGTCATGGAAATGAACAGTTAACTTGGCACCGGATTTTTGGTTATTTTGGACATCA
AAGATATTTTGCATCGTCATTCATGTTAGATCACAGAAAGGCTTTTGGTTCCTATGATCACAAAAATGAC
ATTCACATACGATATGTTTTTTCTTTCTTTCTCTATTTTGTCTACAGGCCCAGCCTACTAGGAGCAAATC
GACTCATCGATCCTTTCATTTATGTTAATCTCATAACTATTGTTCTGGGCGTTACTCTTGCTCAATAAGA
TATTATGCTATTAGCCTAAGACAGTTTGCTTTGTGAGATGGATCTCGTGACTAGGATAAACTCGATACGA
GTCTTGGTCTAGGATCAAGGGTAAAGCCATGGCAGACTCTGTTACTCGAGCCATGTTGCCGGGTCAGTTG
AAATTCATTCGAAATCTGACTGTTGATCTGCTTTTCTATCTACAGAACTCATCAATATA

Figure S1. Schematic representation of *F. graminearum* PH-1 *FgVe1* gene coding sequence with indication of regions, used as targets for dsRNAs: blue for FgVe1_150 dsRNA, yellow for FgVe1_540 dsRNA, green for FgVe1_370 dsRNA. Primers sequences are highlighted.

ATGCACTTAACTGAGGAGATCCTTAAGGAGAATCCAAATCTTTGTGCCTACGAGGCACCATCGTTGGATGCCAGACA
AGACATGGTGGTGGTCGAGGTGCCTAAGTTGGGCAAAGAGGCTGCTACCAAGGCCATCAAGGAATGGGGTCAGCCCA
AGTCGAAGATCACCCACGTGGTCTTTTGCACCACCAGTGGTGTCGACATGCCTGGAGCTGACTACCAGCTCACCAAG
CTATTGGGGCTTCGACCCTCGGTCAAGCGTTTGATGATGTACCAACAAGGATGTTTCGCCGGTGGCACGGTCCTCAG
AGTCGCCA

Figure S2. Schematic representation of *H. lupulus ChS* gene region, used as target for control dsRNAs.

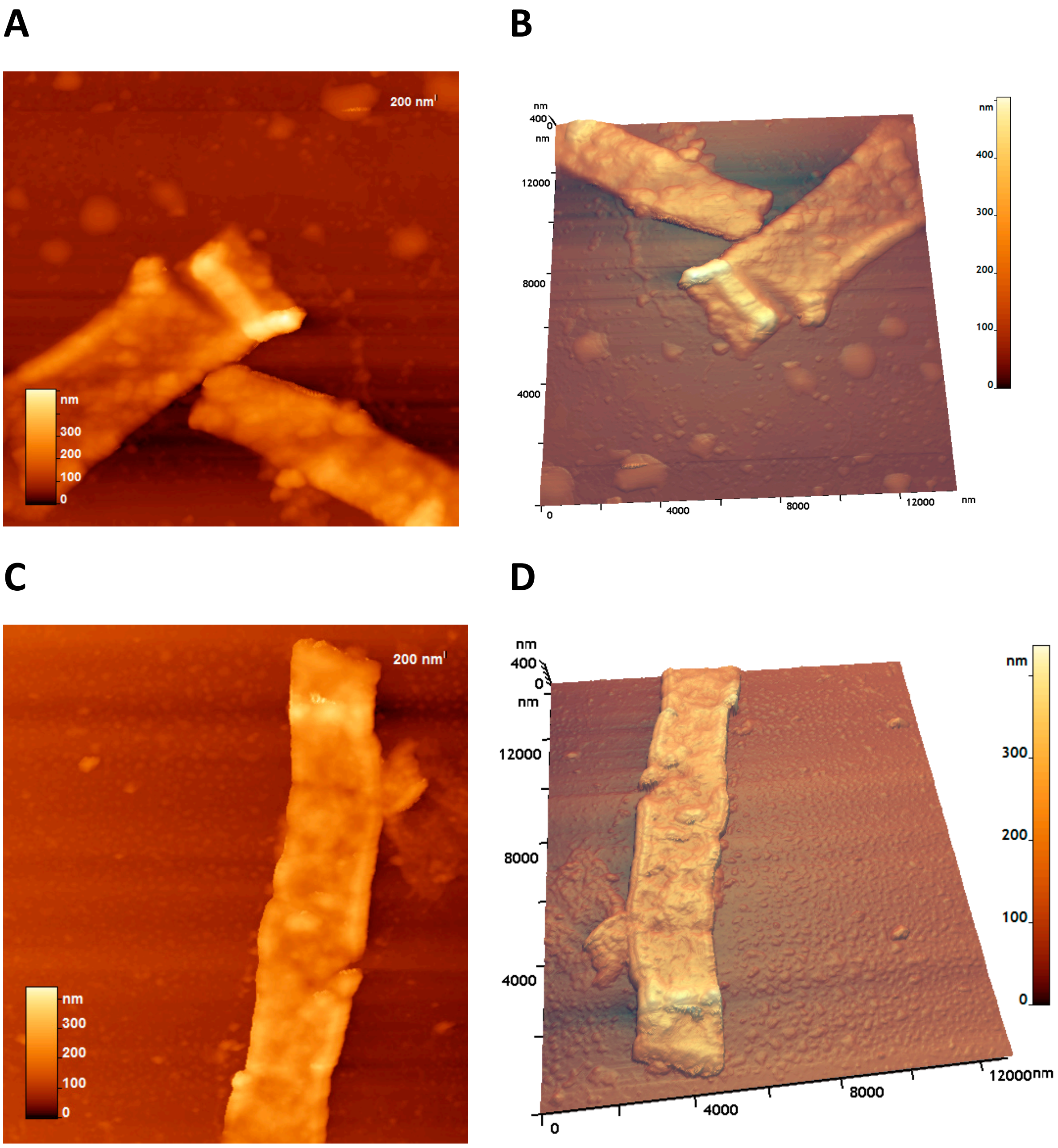
A
200 nm
nm
300
200
100
0
B
nm
400
0
nm
12000
8000
4000
0
4000
8000
12000
nm
400
300
200
100
0
C
200 nm
nm
300
200
100
0
D
nm
400
0
nm
12000
8000
4000
0
4000
8000
12000nm
nm
300
200
100
0

E **F**

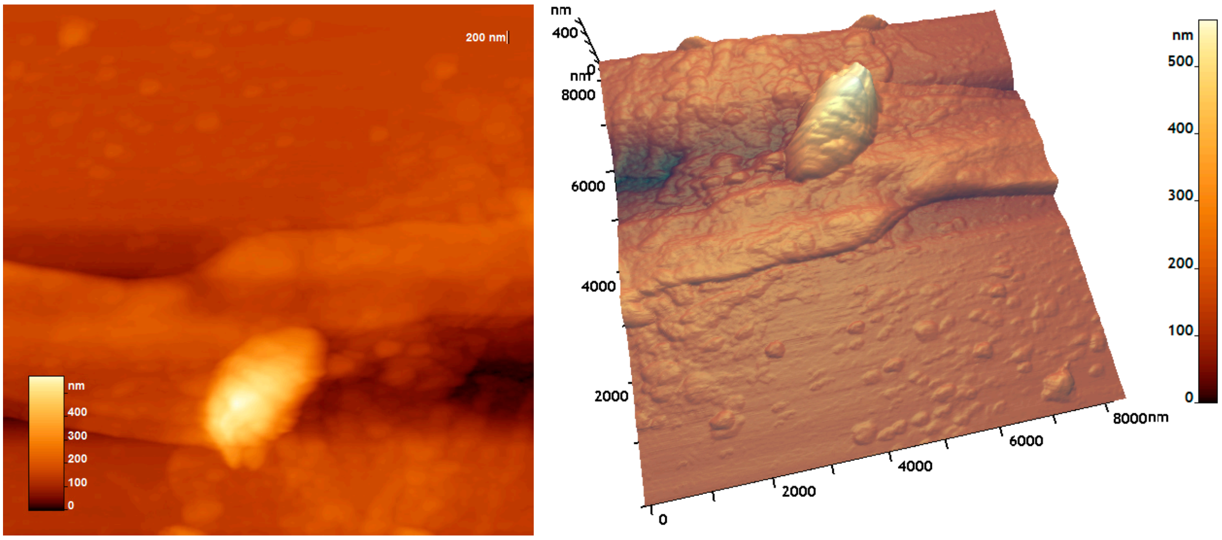

Figure S3. AFM image of *F. graminearum* with CaPs. (A, C, E – 2D image, B, D, F – 3D image). A, B – F. graminearum hyphae on mica surface, C, D – 10 µl of *F. graminearum* and 1 µl of CaPs were mixed in an Eppendorf and applied immediately on mica. E, F – *F. graminearum*+CaPs after exposure for 24 hours in a refrigerator. Particles matching the shape and size of CaP particles are visible on the substrate and hyphae (C,D,E,F). The images shows the adsorption of the particles on hyphae.

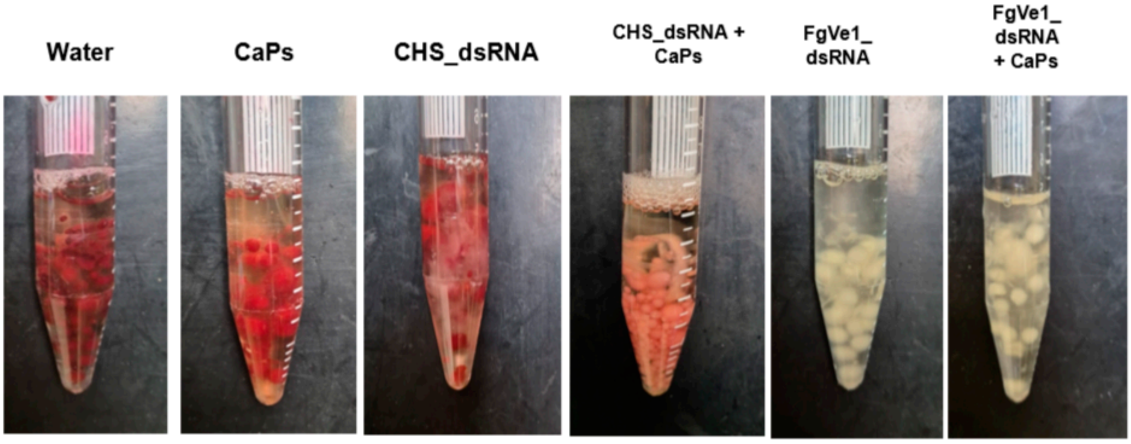

Figure S4. Effect of FgVe1_540 dsRNA and CaP:FgVe1_540 dsRNA nanocomplexes treatment on *F. graminearum* MFG 58918 color in liquid cultures . Photographs were taken at 7 dpi.

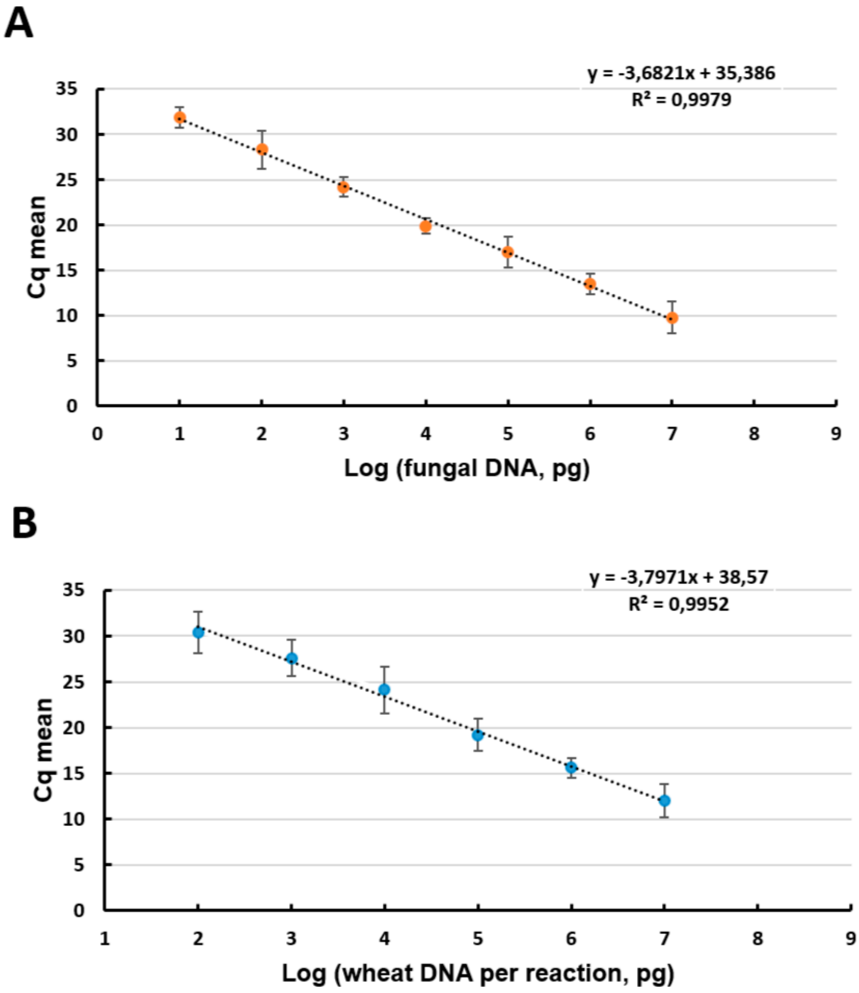

Figure S5. Standard curves, generated using ten-fold dilutions of *Fusarium* (A) and wheat (B) DNA and showing the correlation between quantification cycle (Cq) and DNA copy number (logarithmic). Standard deviations are indicated next to each corresponding point.

Table S1. Oligonucleotides used in relative transcription analysis

Primer sequences, 5'-3'	Probe sequences, 5'-3'	Efficiency	Reference
F: CAGCTTATTCGAGACTACCAAGGAG R: GGGGAACTTCTTGGCGCT	BHQ1-CCTGCCGATGGCGA(FAMdT)CTTCCACCTGG	90%	[77]
F: TGGGCACTYGTCAACG R: ATCCARCATCCCTCRAAAAAG	BHQ1-CCATAGTGCTACGGA(FAMdT)AAGGTTCAATGAGCAG	86%	[77]
F: ACTATGAATCACCAACWTTCGAA R: TTGTGTATCCGCCTATAGTGATC	BHQ1-CAAGGGCACCGCAC(FAMdT)GTTGGTTTGTG	90%	[77]
F: AAGAGACTGCATGGATGACAGC R: GGCTGCGTTCTTGTTATCTCG	BHQ1-TGGCTCACRGCTT(FAMdT)CCTAGGAGCYATGTC	89%	[77]
F: GAGAAYAGAGAGCGCATGATTG R: CGTACATCYAGRACAGTTGCCAT	BHQ1-TTCCGAATCAACAT(FAMdT)TCCAAAGCGAGAA	83%	This study
F: TGGGCACTYGTCAACG R: ATCCARCATCCCTCRAAAAAG	BHQ-CCATAGTGCTACGGA(FAMdT)AAGGTTCAATGAGCAG	90%	[77]
